# Post-thoracotomy intercostal artery pseudoaneurysm manifesting as a chest wall metastasis

**DOI:** 10.1007/s12055-023-01485-9

**Published:** 2023-02-16

**Authors:** Konstantinos Kostopanagiotou, Małgorzata Edyta Wojtyś, Kajetan Kiełbowski, Konstantinos Papagiannopoulos

**Affiliations:** 1 Department of Thoracic Surgery, Attikon University Hospital, Athens, Greece; 2 Department of Thoracic Surgery and Transplantation, Pomeranian Medical University, Alfreda Sokołowskiego 11, 70-891 Szczecin, Poland; 3 Department of Thoracic Surgery, St James’s University Hospital, Leeds, UK

**Keywords:** Intercostal artery pseudoaneurysm, Thoracotomy, Thoracic surgery, Thrombin embolization

## Abstract

Intercostal artery pseudoaneurysm (IAP) represents an extremely rare vascular abnormality developing after an insult to the vascular wall with blood collection within the vascular wall layers and subsequent dilatation. Treatment options, apart from observation, include embolization, endovascular stenting, and surgical correction. We describe the case of a 73-year-old male patient with colonic adenocarcinoma pulmonary metastasis. Repetitive wedge resections and a right lower lobectomy were performed to remove multiple metastatic lesions. At follow-up assessment, the patient reported localized thoracotomy site pain progressing with time and unresponsive to oral analgesics. Chest computed tomography (CT) revealed a pseudoaneurysm of 4-cm diameter of the right 5^th^ intercostal artery. The patient underwent embolization of the lumen and was discharged from the hospital after 24 h. Successive CT re-assessment checks were unremarkable.

## Introduction

Pseudoaneurysms of the intercostal arteries (IAP) are extremely rare vascular entities. These develop due to vascular wall insults secondarily to trauma or percutaneous interventions where blood collects within the vascular wall layers [1]. Distinctively different, true aneurysms are full-thickness dilated arteries mostly associated with genetic diseases such as neurofibromatosis type 1. As the intercostal arteries originate from the posterior intercostal arteries stemming out from the aorta (anterior intercostal arteries originate from the internal thoracic arteries), rupture with arterial bleeding is a catastrophic risk. Limited available information exists on their management. In this paper, we present an IAP case developing after repetitive thoracotomies, strongly resembling metastasis and treated by thrombin embolization. Successive follow-up checks were unremarkable.

## Case report

**Fig. 1:**
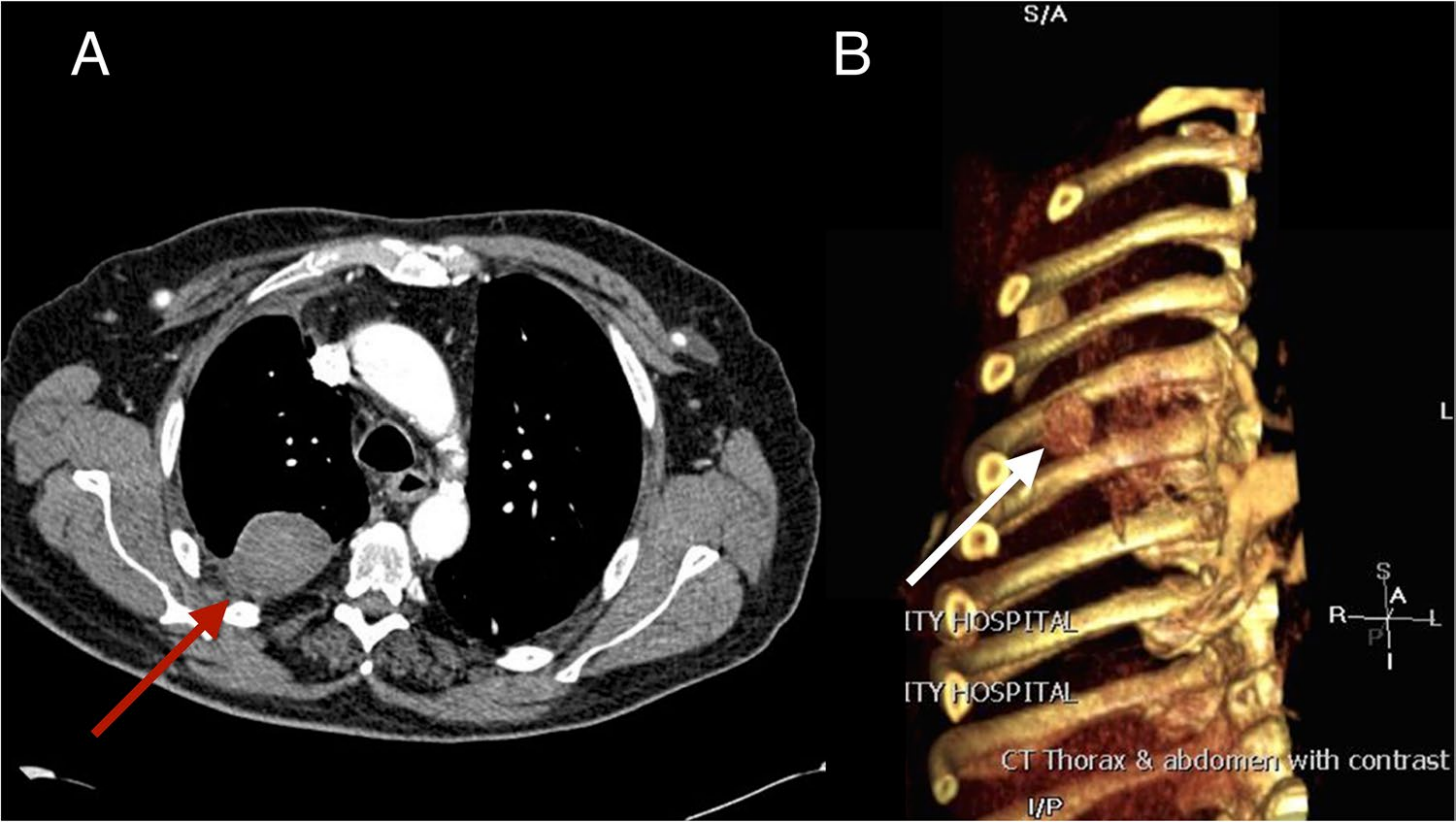
Abnormal contrast-enhanced thoracic computerized tomography (**A**) and 3-dimensional image (**B**) showing the 4-cm pseudoaneurysm of the right 5.^th^ intercostal artery (red and white arrows)

**Fig. 2:**
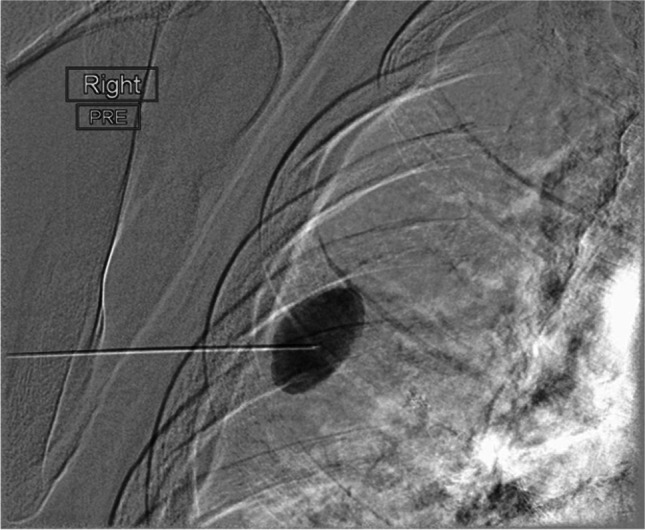
The intercostal pseudoaneurysm section is percutaneously embolized with 3.5 ml of thrombin directly in the lumen under local anesthetic in the computerized tomography (CT) suite

A 73-year-old male patient developed bilateral pulmonary metastases a year after a sigmoidectomy for adenocarcinoma. The right-sided lesions were managed once by video-assisted thoracoscopic surgery (VATS) and twice by posterolateral thoracotomy incisions (5^th^ intercostal space) for repetitive wedge resections, including a lower lobectomy for a centrally located metastasis. These surgical procedures were uneventful. After three postoperative months, he complained of increasing localized thoracotomy site pain of grades 4–5 at visual analogue scale (VAS), non-responding to oral analgesics. The chest X-ray at 3 months showed an abnormal round opacity of the right middle zone. Differential diagnosis included mainly metastasis and infection which necessitated further imaging investigations. Contrast chest computed tomography (CT) identified a 4-cm diameter pseudoaneurysm of the right 5^th^ intercostal artery at the lateral thoracotomy level (Fig. 1). Management options for this lesion included observation with rescanning, surgical aneurysmectomy, or radiologic embolization. After discussion with the patient and considering the bleeding risk and disabling pain, we selected the least invasive treatment option of embolization. The interventional radiologist successfully injected in the lumen 3.5 ml of thrombin under CT guidance obliterating the IAP (Fig. 2). The patient remained 24 h in hospital for observation. Repetitive CT scans prior to discharge and at 6 months were unremarkable.

## Discussion

This extremely rare finding is commonly the result of iatrogenic vascular injury, i.e., sternotomy, thoracoscopy, thoracentesis, percutaneous lung biopsy, and even after transaortic transcatheter aortic valve implantation (TAVI) [1, 2]. It is even observed after liver biopsy, biliary procedures, or nephrectomy [1]. Furthermore, non-procedure–related IAPs may present as acute back pain [3]. Symptoms are largely nonspecific, including acute or chronic pain of increasing intensity, dyspnea, or presence of a pulsatile thoracic mass [4]. The non-specific symptomatology may cause confusion to an undiscerning physician leading to misdiagnosis of chronic pain and mistreatment with long-term analgesics. Abnormal chest X-rays may resemble metastasis, leading to a diagnostic contrast CT study. A rupture may lead to life-threatening hemothorax requiring immediate hemorrhage control interventions. Hemomediastinum is rare and was observed only in one patient [3]. The Adamkiewicz artery is the largest anterior radiculomedullary artery which originates from the posterior branch of the intercostal artery. It enters the spinal canal and supplies several segments of the spinal cord. Damage or thrombosis of the vessel may cause infarction of the spinal cord. Therefore, it is crucial to identify the Adamkiewicz artery prior to any intervention to prevent serious potential neurologic complications [5]. The main treatment options for IAPs, as described in case reports, include stent insertion, observation, aneurysmectomy [6], and embolization. Thrombin is a common embolic material for intravascular application. It stimulates thrombus formation by activating fibrinogen with minimal side effects. Alternatively, endovascular coil embolization could be considered an option.

## Conclusions

We conclude that IAPs are rare but potentially life-threatening and commonly misdiagnosed and mistreated. A suspicion should exist in postoperative thoracic patients with persisting thoracotomy pain and abnormal imaging, particularly in CT contrast scans. Percutaneous thrombin embolization should be the preferred treatment over surgery.
